# Dietary polyphenol intake and risk of type 2 diabetes in the Polish arm of the Health, Alcohol and Psychosocial factors in Eastern Europe (HAPIEE) study

**DOI:** 10.1017/S0007114517001805

**Published:** 2017-07-14

**Authors:** Giuseppe Grosso, Urszula Stepaniak, Agnieszka Micek, Magdalena Kozela, Denes Stefler, Martin Bobak, Andrzej Pajak

**Affiliations:** 1 Integrated Cancer Registry of Catania-Messina-Siracusa-Enna, Via S. Sofia 85, 95123, Catania, Italy; 2 Department of Epidemiology and Population Studies, Jagiellonian University Medical College, 20 Grzegorzecka Street, Krakow 31���531, Poland; 3 Department of Epidemiology and Public Health, University College London, 1–19 Torrington Place, London WC1E 6BT, UK

**Keywords:** Diabetes, Polyphenols, Flavonoids, Phenolic acids, Lignans, Stilbenes, Prospective studies

## Abstract

This study aimed to test the association between dietary content of total and individual classes of polyphenols and incident cases of type 2 diabetes in Polish adults participating to the Health, Alcohol and Psychosocial factors In Eastern Europe study. At baseline, diet by 148-item FFQ and health information were collected from 5806 participants free of diabetes. Self-reported incident type 2 diabetes was ascertained at 2–4-year follow-up visit. OR and 95 % CI of type 2 diabetes comparing the various categories of polyphenol intake to the lowest one (reference category) and as 1 sd increase modelled as continuous variable were calculated by performing age-, energy-, and multivariate-adjusted logistic regression models. During the follow-up, 456 incident cases of type 2 diabetes occurred. When comparing extreme quartiles, intake of total polyphenol was inversely associated with the risk of type 2 diabetes (OR 0·43; 95 % CI 0·30, 0·61); 1 sd increase was associated with a reduced risk of diabetes (OR 0·68; 95 % CI 0·59, 0·79). Among the main classes of polyphenols, flavonoids, phenolic acids, and stilbenes were independent contributors to this association. Both subclasses of phenolic acids were associated with decreased risk of type 2 diabetes, whereas among subclasses of flavonoids, high intake of flavanols, flavanones, flavones and anthocyanins was significantly associated with decreased risk of type 2 diabetes. Total dietary polyphenols and some classes of dietary polyphenols were associated with lower risk of type 2 diabetes.

Plant-derived dietary pattern have been suggested to exert protection against metabolic disorders, such as type 2 diabetes^(^ ^1^ ^)^. Among the most attractive hypotheses potentially explaining the benefits associated with high consumption of fruits and vegetables, polyphenols content is one of the best candidates as determinant of health. In fact, besides the known macro- and micronutrients contained in fruit and vegetables, polyphenols are highly representative in beverages such as coffee, tea and some alcoholic beverages that have demonstrated to have a positive impact on human health^(^ ^2^ ^)^. Polyphenolic compounds are molecules highly contained in the aforementioned food and plant-derived beverages divided into four main classes on the basis of their chemical structure, including flavonoids, phenolic acids, stilbenes, and lignans, and ‘other’ polyphenols including various type of compounds^(^ ^3^ ^)^. Overall, polyphenols comprise thousands of different molecules have been classified and described. Even though, the main challenges of studying these compounds rely on the identification of their potential effects in humans. *In vitro* studies demonstrate consistently antioxidant and anti-inflammatory effects at cellular and tissue level, but questions remain regarding their effective relevance for human health, as exposure in normal diet occur at lower concentrations than in laboratory setting^(^ ^3^ ^,^ ^4^ ^)^.

Although causality is best supported by randomised trials, observational epidemiological studies also contribute to causal inference and are of great interest to evaluate the ‘real world’ consumption of polyphenols and their potential association with health outcomes. Regarding metabolic disorders, type 2 diabetes has been one of the most studied outcome evaluated in epidemiological studies. A relatively recent meta-analysis showed that increased intake of flavonoids, especially flavonols, may decrease the risk of type 2 diabetes^(^ ^5^ ^)^. However, available data on type 2 diabetes prevalence and incidence is focused only on flavonoids. Only recently research has expanded to other polyphenol groups, such as phenolic acids and individual subgroups but data are overall scarce. Moreover, as polyphenol consumption varies with a great extent with the dietary habits of a population, it is of interest to collect information from various countries in order to provide stronger evidence of their potential effects. In this study, we aimed to test the association between dietary content of total and individual classes of polyphenols and self-reported incident cases of type 2 diabetes in Polish adults participating to the Health, Alcohol and Psychosocial factors in Eastern Europe (HAPIEE) study.

## Methods

### Study population

The HAPIEE study is a multicenter prospective cohort study investigating the role of biological, dietary, lifestyle and environmental factors in cardiovascular and other chronic diseases^(^ ^6^ ^)^. Information on methods used are reported in detail elsewhere^(^ ^6^ ^)^. The present study was conducted on a subcohort of the Polish arm of the HAPIEE cohort (a random sample of 10 728 adults aged 45–69 years recruited in 2002–2005 (response ratio of 59 %) in the urban area of Krakow, Poland) which was free of diabetes or impaired fasting glucose (fasting plasma glucose from 6·1 to 6·9 mmol/l) at baseline (*n* 9420). Study participants completed a structured questionnaire and underwent to a clinical examination during the baseline data collection (wave I) and at follow-up visit (wave II). Participants were followed for a median follow-up period of 4 years (range 3·2–5·4 years). For the purpose of this study, only individuals free of diabetes at baseline who attended the last follow-up visit in 2006–2008 were included in the analysis (*n* 5806, 54 % of original cohort). Sample included in the analysis did not substantially differ in background characteristics or variables of interest concerning dietary habits. All participants provided written informed consent before joining the study.

### Dietary assessment

Dietary data were collected by using a FFQ previously validated^(^ ^7^ ^,^ ^8^ ^)^. The FFQ consisted of 148 food and drink items accompanied by a country-specific instruction manual that included photographs to facilitate the estimation of portion sizes. Participants were asked how often, on average, they had consumed that amount of the item during the last 3 months, with nine responses ranging from ‘never or less than once per month’ to ‘six or more times per day’. Participants were also asked to include additional foods and frequency of consumption by manual entry.

### Estimation of polyphenol intake

Data on the polyphenol content in foods were obtained from the Phenol-Explorer database (www.phenol-explorer.eu)^(^ ^9^ ^)^. The process of estimation of polyphenol intake has been described in details elsewhere^(^ ^10^ ^)^. In brief, food items of the FFQ containing more food components were separated according to their ingredients and foods that contained no polyphenols were excluded from the analysis. The average food consumption was calculated (in g or ml) by following the standard portion sizes used in the study and then converted in 24-h intake. An advanced search was carried out in the Phenol-Explorer database to retrieve mean content values for all polyphenols contained in the foods obtained and individual polyphenol intake from each food was calculated by multiplying the content of each polyphenol by the daily consumption of each food. Total polyphenol intake was calculated as the sum of all individual polyphenol intake from all food sources encountered according to this process. In this study we investigated exposure to total polyphenols and their main classes phenolic acids, flavonoids, stilbenes and lignans; the main subclasses of phenolic acids, including hydroxybenzoic acids and hydroxycinnamic acids; the main subclasses of flavonoids, including flavanols, flavonols, flavanones, flavones, anthocyanins, and isoflavones; and ‘other’ polyphenols, including alkylmethoxyphenols, alkylphenols, curcuminoids, furanocoumarins, hydroxybenzaldehydes, hydroxybenzoketones, hydroxycinnamaldehydes, hydroxycoumarins, hydroxyphenylpropenes, methoxyphenols, naphtoquinones, phenolic terpenes and tyrosols.

### Demographic, lifestyle and clinical measurements

Socio-demographic and lifestyle characteristics included age, sex, educational and occupational level, smoking and alcohol drinking habits. Physical activity included energy expenditure in leisure time by reporting type and duration of activity according the predetermined questionnaire items. The overall amount of energy expenditure was estimated in kJ (kcal/d) and categorised in low, moderately and high activity level. Individuals were categorised according their smoking status as non-smoker and current smoker. Alcohol consumption was categorised as (i) non-drinkers (ii) drinkers; alcohol intake was considered as a continuous variable (g/d).

Physical examination included measurement of height, weight, waist circumference and blood pressure using standard procedures^(^ ^6^ ^)^. BMI was calculated according to the formula weight (kg)/height (m^2^).

### Outcome assessment

Participants were considered to have type 2 diabetes at baseline if they had elevated glucose concentrations (plasma glucose concentrations of ≥7 mmol/l) or treatment with hypoglycaemia medications (insulin or oral hypoglycaemia agent) within the last 2 weeks. Among individuals free of diabetes at baseline, cases of new type 2 diabetes were defined as those participants self-reporting of being professionally diagnosed with type 2 or taking hypoglycaemia medications within the last two weeks at follow-up.

### Statistical analysis

We categorised individual flavonoids by dividing intake into quartiles and providing mean intakes for each category of exposure (expressed as glycosides and esters). Characteristics of the study cohort were described by quartiles of total polyphenol consumption. Sex-specific analyses were conducted. Descriptive presentation relied on cross tabulations. Continuous variables are presented as means and standard deviations, categorical variables as counts and percentages. Variables were examined for normality (Kolmogorov test). The *χ* ^2^ test was used for comparisons of categorical variables, the Kruskal–Wallis test was used for continuous variables because these variables did not fit a normal distribution.

The association between baseline consumption of polyphenols and incident type 2 diabetes was assessed by logistic regression analyses. Age- and energy-adjusted, and multivariable-adjusted models were performed: OR and 95 % CI of having type 2 diabetes were calculated for polyphenols considered as both categorical (quartiles, with the lower category of polyphenol consumption as reference) and continuous exposure (1 sd increase intake). Tests for linear trends were also performed by assigning the medians of each quartile as scores. Variables included in the multivariable model were age, total energy intake, BMI, physical activity, educational status, smoking status, alcohol consumption, alcohol intake, fibre and menopausal status (women only). As Spearman correlation test showed significant correlation between main classes of polyphenols and subclasses of flavonoids (data not shown), we did not include in the model all main classes of polyphenols/flavonoids. Graphical representation using restricted cubic splines analysis for total polyphenols was applied. When examining the association with total polyphenols, a sensitivity analysis was performed by including one at the time the major food sources of polyphenols based on our previous publication^(^ ^10^ ^)^, to test whether the level of association was driven by one individual food component. Statistical significance was accepted at *P*<0·05. All statistical analyses were performed with SPSS for Windows 21.0 (SPSS Inc.).

## Results

Baseline characteristics of individuals free of diabetes by quartiles of total polyphenol intake are presented in Table 1. Age and BMI were significantly lower with increasing polyphenol intake. In contrast, energy intake, fibre, alcohol drinkers and alcohol intake increased through quartiles of polyphenol consumption. Finally, among individuals with higher intake of polyphenols there were more smokers and physically active (Table 1). Major food contributors for total polyphenol intake were coffee and tea, representing the major contributors of phenolic acid and flavonoid classes, respectively (online Supplementary Table S1). Among fruits and vegetables, other important contributors of flavonoids were black currant, apples, strawberries, beans and oranges. Regarding lignans and stilbenes, major food sources were seeds and red wine, respectively; ‘other’ polyphenols were mainly contained in beer, cereals, and coffee (online Supplementary Table S1).

**Table 1:** Background characteristics of participants in the Health, Alcohol and Psychosocial factors in Eastern Europe cohort free of diabetes at baseline by quartiles (Q) of total polyphenol intake (energy adjusted) (Numbers and percentages; mean values and standard deviations)

	Polyphenol quartiles (men)								
	Q1		Q2		Q3		Q4		
	*n*	%	*n*	%	*n*	%	*n*	%	*P*_for trend_
No. of subjects	1347		1454		1481		1524		
Age (years)									<0·001
Mean	57·7		57·9		57·3		56·6		
sd	6·9		6·9		6·8		6·8		
Men	645	47·9	656	45·1	698	47·1	732	48·0	0·632
BMI (kg/m^2^)									<0·001
Mean	28·1		27·8		27·8		27·3		
sd	4·3		4·3		4·3		4·1		
Current smoker	409	30·5	451	31·0	454	30·8	528	34·7	0·022
Educational level									0·056
Low	135	10·1	162	11·2	143	9·7	134	9·9	
Medium	818	61·0	859	59·2	895	60·4	878	59·5	
High	389	29·0	430	29·6	443	33·6	512	30·6	
Physical activity level									0·010
Low	385	30·1	401	29·3	377	26·8	398	27·4	
Medium	483	37·8	517	37·7	534	37·9	530	36·6	
High	409	32·0	452	33·0	498	35·3	522	36·0	
Alcohol drinkers	582	43·2	743	51·1	820	55·4	858	56·3	<0·001
Alcohol intake (g/d)									<0·001
Mean	2·16		2·34		2·99		3·31		
sd	5·64		5·73		7·01		9·23		
Total energy intake (kJ/d)									<0·001
Mean	7524		8490·50		9345·80		10 335·65		
sd	2204·29		2178·35		2449·43		2833·32		
Total energy intake (kcal/d)									<0·001
Mean	1798·28		2029·28		2233·70		2470·28		
sd	526·84		520·64		585·43		677·18		
Fibre intake (mg/d)									<0·001
Mean	15·85		18·02		19·70		22·17		
sd	5·53		5·94		7·18		8·94		

**Fig. 1:**
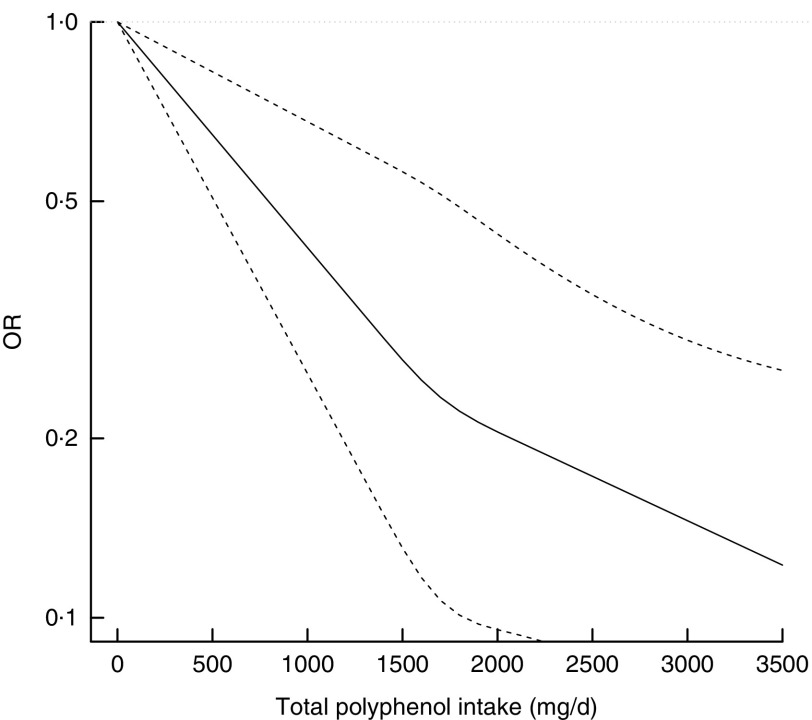
Association between total polyphenol intake and incidence of type 2 diabetes.

During follow-up, 456 incident cases of type 2 diabetes occurred. In fully adjusted analyses, the highest intake of total polyphenols was associated with lower risk of type 2 diabetes in the whole population (Table 2) and individually in both men and women (OR 0·31; 95 % CI 0·18, 0·52 and OR 0·53; 95 % CI 0·33, 0·85, respectively; online Supplementary Table S2) compared with the lowest. The relation was linear (Fig. 1) and 1 sd increased intake of polyphenols was associated with a 32 % reduced risk of diabetes (OR 0·68; 95 % CI 0·59, 0·79). The sensitivity analysis by adjusting for major food sources of polyphenols showed no differences with previous results (data not shown). Among the main classes of polyphenols, flavonoids, phenolic acids and stilbenes were contributors to this association. When considering individual subclass contributors among phenolic acids, both hydroxybenzoic acids and hydroxycynnamic acids showed an inverse relation with type 2 diabetes in a linear dose–response manner (Table 3). However, separate analyses by sex showed that higher intake of hydroxybenzoic acids was associated with decreased risk of type 2 diabetes mainly in men (online Supplementary Table S3). Among individual subclasses of flavonoids, flavanols, flavanones, flavones and anthocyanins were independently associated with decreased risk of type 2 diabetes in a linear dose–response manner (Table 4). Analyses separated by sexes showed similar results on flavones, whereas most of other results were more evident in men (online Supplementary Table S4). The association between other polyphenols and risk of type 2 diabetes resulted in null results for the whole cohort (Table 5) and individually for men and women (online Supplementary Table S5).

**Table 2:** Association between cumulative polyphenol intake (total and main groups) and incidence of type 2 diabetes (Mean values and standard deviations; odds ratios and 95 % confidence intervals)

	Polyphenol quartiles										
	Q1		Q2		Q3		Q4			1 sd increase	
	OR	95 % CI	OR	95 % CI	OR	95 % CI	OR	95 % CI	*P*_for trend_	OR	95 % CI
Total polyphenols (mg/d)											
Mean	1026·7		1469·6		1872·6		2632·1				
sd	212·0		102·2		136·7		608·0				
No. of cases	167		121		91		77				
Model 1*	1		0·69	0·54, 0·89	0·54	0·41, 0·71	0·47	0·35, 0·64	<0·001	0·72	0·63, 0·81
Model 2†	1		0·77	0·58, 1·01	0·48	0·35, 0·66	0·43	0·30, 0·61	<0·001	0·68	0·59, 0·79
Total flavonoids (mg/d)											
Mean	501·8		756·4		958·2		1452·3				
sd	125·1		55·6		69·8		509·4				
No. of cases	165		122		92		77				
Model 1*	1		0·76	0·59, 0·98	0·57	0·43, 0·75	0·53	0·39, 0·72	<0·001	0·77	0·67, 0·89
Model 2†	1		0·75	0·56, 0·99	0·50	0·36, 0·68	0·44	0·30, 0·63	<0·001	0·72	0·61, 0·84
Phenolic acids (mg/d)											
Mean	288·5		615·8		829·3		1482·0				
sd	85·5		60·6		162·9		298·5				
No. of cases	126		137		114		79				
Model 1*	1		1·03	0·80,1·33	0·95	0·73,1·25	0·64	0·47,0·86	<0·001	0·80	0·72, 0·90
Model 2†	1		1·13	0·85, 1·50	0·92	0·68, 1·25	0·60	0·42, 0·84	0·001	0·80	0·71, 0·91
Stilbenes (mg/d)											
Mean	0·004		0·016		0·046		0·598				
sd	0·003		0·005		0·013		1·336				
No. of cases	169		102		107		78				
Model 1*	1		0·60	0·46, 0·78	0·62	0·48, 0·81	0·40	0·30, 0·54	<0·001	0·90	0·75, 1·07
Model 2†	1		0·59	0·44, 0·79	0·54	0·40, 0·73	0·34	0·24, 0·48	<0·001	0·92	0·77, 1·11
Lignans (mg/d)											
Mean	0·16		0·24		0·31		1·36				
sd	0·04		0·01		0·02		19·55				
No. of cases	121		103		114		118				
Model 1*	1		0·98	0·74, 1·30	1·11	0·84, 1·45	1·25	0·95, 1·65	0·672	0·98	0·87, 1·11
Model 2†	1		0·98	0·71, 1·34	1·15	0·85, 1·56	1·18	0·86, 1·61	0·534	1·00	0·88, 1·13

Q, quartile.

* Adjusted for age (continuous) and total energy intake (continuous).

† Model 1+adjusted for BMI (continuous), physical activity (low/medium/high), educational status (low/medium/high), smoking status (yes/no), alcohol consumption (yes/no), alcohol intake (continuous), menopausal status (women only, yes/no) and dietary fibre (continuous).

**Table 3:** Association between phenolic acid subclasses and incidence of type 2 diabetes (Mean values and standard deviations; odds ratios and 95 % confidence intervals)

	Polyphenol quartiles										
	Q1		Q2		Q3		Q4			1 sd increase	
	OR	95 % CI	OR	95 % CI	OR	95 % CI	OR	95 % CI	*P*_for trend_	OR	95 % CI
Hydroxybenzoic acids (mg/d)											
Mean	43·4		85·7		91·9		157·1				
sd	23·5		0·9		3·7		31·4				
No. of cases	124		118		128		76				
Model 1*	1		0·93	0·71, 1·21	1·19	0·91, 1·56	0·75	0·56, 1·00	0·115	0·92	0·83, 1·02
Model 2†	1		1·05	0·78, 1·42	1·18	0·87, 1·60	0·71	0·51, 0·99	0·043	0·89	0·79, 0·99
Hydroxycinnamic acids (mg/d)											
Mean	188·19		530·67		724·48		1386·85				
sd	73·6		66·5		168·8		292·2				
No. of cases	127		128		121		80				
Model 1*	1		0·95	0·73, 1·23	1·01	0·77, 1·32	0·64	0·47, 0·85	<0·001	0·81	0·73, 0·91
Model 2†	1		1·08	0·81, 1·44	0·99	0·73, 1·34	0·61	0·43, 0·86	0·001	0·80	0·71, 0·91

Q, quartile.

* Adjusted for age (continuous) and total energy intake (continuous).

† Model 1+adjusted for BMI (continuous), physical activity (low/medium/high), educational status (low/medium/high), smoking status (yes/no), alcohol consumption (yes/no), alcohol intake (continuous), menopausal status (women only, yes/no) and dietary fibre (continuous).

**Table 4:** Association between flavonoid subclasses and incidence of type 2 diabetes (Mean values and standard deviations; odds ratios and 95 % confidence intervals)

	Polyphenol quartiles										
	Q1		Q2		Q3		Q4			1 sd increase	
	OR	95 % CI	OR	95 % CI	OR	95 % CI	OR	95 % CI	*P*_for trend_	OR	95 % CI
Flavanols (mg/d)											
Mean	323·65		519·72		681·84		1099·91				
sd	109·97		57·01		43·89		434·73				
No. of cases	151		139		86		80				
Model 1*	1		0·96	0·75, 1·23	0·60	0·45, 0·80	0·59	0·43, 0·79	<0·001	0·77	0·67, 0·88
Model 2†	1		0·88	0·67, 1·17	0·59	0·43, 0·81	0·54	0·39, 0·76	0·001	0·77	0·66, 0·90
Flavonols (mg/d)											
Mean	60·16		89·63		113·64		163·31				
sd	14·77		6·35		7·71		46·28				
No. of cases	124		114		110		108				
Model 1*	1		1·00	0·77, 1·31	1·01	0·77, 1·33	1·12	0·84, 1·49	0·275	1·05	0·95, 1·16
Model 2†	1		1·00	0·73, 1·35	0·98	0·72, 1·34	0·89	0·63, 1·26	0·368	0·94	0·82, 1·07
Flavanones (mg/d)											
Mean	24·68		59·64		105·55		218·88				
sd	11·44		11·05		15·86		89·61				
No. of cases	134		126		98		98				
Model 1*	1		0·92	0·71, 1·19	0·79	0·60, 1·05	0·85	0·64, 1·13	0·178	0·92	0·82, 1·03
Model 2†	1		0·88	0·66, 1·18	0·73	0·53, 1·00	0·75	0·54, 1·03	0·047	0·88	0·77, 0·99
Flavones (mg/d)											
Mean	1·96		4·28		7·93		16·53				
sd	0·70		0·77		1·56		7·71				
No. of cases	144		123		104		85				
Model 1*	1		0·80	0·62, 1·04	0·74	0·56, 0·97	0·68	0·50, 0·92	0·188	0·92	0·81, 1·04
Model 2†	1		0·71	0·53, 0·95	0·65	0·47, 0·89	0·47	0·33, 0·68	0·021	0·84	0·72, 0·97
Anthocyanins (mg/d)											
Mean	4·40		8·75		14·52		91·92				
sd	2·02		1·24		2·46		185·59				
No. of cases	114		141		113		88				
Model 1*	1		1·32	1·01, 1·71	1·08	0·82, 1·42	0·87	0·65, 1·18	0·257	0·89	0·74, 1·08
Model 2†	1		1·18	0·88, 1·59	0·86	0·62, 1·19	0·68	0·48, 0·98	0·070	0·82	0·66, 1·01
Isoflavones (mg/d)											
Mean	0·001		0·140		0·196		4·912				
sd	0·001		0·001		0·001		13·659				
No. of cases	74		145		131		106				
Model 1*	1		0·91	0·68, 1·22	1·04	0·77, 1·41	0·90	0·65, 1·23	0·211	0·85	0·67, 1·09
Model 2†	1		0·91	0·65, 1·28	1·00	0·71, 1·40	0·93	0·65, 1·34	0·065	0·75	0·55, 1·01
Dihydrochalcones (mg/d)											
Mean	1·94		6·93		9·77		25·02				
sd	1·22		1·69		1·09		7·74				
No. of cases	94		130		88		144				
Model 1*	1		1·33	1·00, 1·76	1·05	0·77, 1·43	1·61	1·21, 2·12	0·004	1·15	1·04, 1·26
Model 2†	1		1·07	0·78, 1·46	0·84	0·59, 1·19	1·12	0·80, 1·57	0·926	0·99	0·88, 1·12

Q, quartile.

* Adjusted for age (continuous) and total energy intake (continuous).

† Model 1+adjusted for BMI (continuous), physical activity (low/medium/high), educational status (low/medium/high), smoking status (yes/no), alcohol consumption (yes/no), alcohol intake (continuous), menopausal status (women only, yes/no) and dietary fibre (continuous).

**Table 5:** Association between other polyphenols and incidence of type 2 diabetes (Mean values and standard deviations; odds ratios and 95 % confidence intervals)

	Polyphenol quartiles										
	Q1		Q2		Q3		Q4			1 sd increase	
	OR	95 % CI	OR	95 % CI	OR	95 % CI	OR	95 % CI	*P*_for trend_	OR	95 % CI
Others (mg/d)											
Mean	6·17		16·8		32·92		76·66				
sd	3·02		3·10		5·65		37·81				
No. of cases	107		103		127		119				
Model 1*	1		0·92	0·69, 1·22	1·17	0·89, 1·53	1·22	0·93, 1·62	0·217	1·05	0·96, 1·15
Model 2†	1		0·94	0·68, 1·29	1·11	0·82, 1·50	1·11	0·81, 1·53	0·821	1·01	0·91, 1·12

Q, quartile.

* Adjusted for age (continuous) and total energy intake (continuous).

† Model 1+adjusted for BMI (continuous), physical activity (low/medium/high), educational status (low/medium/high), smoking status (yes/no), alcohol consumption (yes/no), alcohol intake (continuous), menopausal status (women only, yes/no), dietary fibre (continuous) and all main classes of flavonoids included in the table (quartiles).

## Discussion

In this study we observed that individuals with higher total dietary polyphenol intake were less likely to develop type 2 diabetes compared with those in the lowest quartile of intake. Among individual classes studied, hydroxycinnamic acids and hydroxybenzoic acids among phenolic acids, and flavanols, flavanones, flavones and anthocyanins among flavonoids resulted to be associated with lower risk of type 2 diabetes.

Flavonoids are mainly contained in fruit, vegetable and tea, which have been associated with benefits toward metabolic disorders^(^ ^11^ ^,^ ^12^ ^)^. These results are consistent with previous findings of the cross-sectional analysis of baseline data from the same cohort in which we found that the same classes of flavonoids were associated with lower odds of having impaired glucose metabolism^(^ ^13^ ^)^. Such findings may explain the results of previous observations showing better outcomes related with plant-based dietary patterns in the HAPIEE cohort^(^ ^14^ ^–^ ^16^ ^)^. Recent prospective studies reported certain benefits for higher intakes of anthocyanins^(^ ^17^ ^)^, flavanols and flavonols^(^ ^18^ ^,^ ^19^ ^)^, despite contrasting results have been also published^(^ ^20^ ^,^ ^21^ ^)^. Findings from meta-analyses regarding the potential benefits of flavonoids on type 2 diabetes^(^ ^5^ ^)^ and glycaemic control^(^ ^22^ ^)^ also provided evidence of association between some flavonoid classes and decreased risk of type 2 diabetes. From a mechanistic point of view, flavonoids may reduce biological pathways related to the development of type 2 diabetes by improving endothelial function, which has been correlated with insulin resistance^(^ ^23^ ^)^. Moreover, experimental studies *in vitro* and *in vivo* demonstrated that flavonoids interact with molecular targets and affect signalling pathways resulting in improvement of glycaemia and suppression of gluconeogenesis^(^ ^24^ ^–^ ^26^ ^)^. Despite we found some null results regarding the flavonol class, which has been previously related with health benefits associated with tea consumption^(^ ^27^ ^)^, some limitations of our study, including reverse causation and genetics related to high tea consumption may explain our unexpected findings.

In this study, hydroxybenzoic and hydroxycinnamic acids, the main subclasses of phenolic acids, were both inversely associated with the risk of type 2 diabetes. Among the most studied hydroxycinnmic acids, chlorogenic acids (CGA) which are contained in coffee, have been reported to exert beneficial effects towards cardiovascular risk factors and metabolic disorders^(^ ^28^ ^)^. We previously showed in the HAPIEE cohort the association between coffee and metabolic disorders, including type 2 diabetes^(^ ^29^ ^)^. Meta-analyses of observational studies conducted on coffee reported significant decreased risk of type 2 diabetes and general metabolic disorders^(^ ^12^ ^,^ ^30^ ^)^. Together with their antioxidant properties, CGA have been found to exert direct effects in regulating glucose metabolism^(^ ^31^ ^)^. CGA have been hypothesised to exert effects on glucose metabolism through their specific competitive inhibition of the glucose-6-phosphate translocase, which in turn inhibit gluconeogenesis, and activation of AMP-activated protein kinase, a sensor and regulator of cellular energy balance that may lead to suppression of hepatic glucose production and fatty acid synthesis^(^ ^32^ ^)^.

Studies on stilbenes have been mostly focused on the effects of resveratrol on cardio-metabolic health^(^ ^33^ ^)^. A recent meta-analysis on resveratrol treatment as an adjunct to pharmacological management in type 2 diabetes mellitus reported a significant reduction of fasting glucose, insulin, glycated Hb (HbA1c) and insulin resistance levels in participants with type 2 diabetes^(^ ^34^ ^)^ but did not affect glycaemic measures in non-diabetic individuals^(^ ^35^ ^)^. We reported that high total stilbenes intake was related with decreased incidence of type 2 diabetes, thus confirming earlier findings derived from clinical setting. The main effects of stilbenes in human health are relative to their role in cellular defense against oxidative stress through the nuclear factor-erythroid-2-related factor-2 (Nrf2) and the potential roles of SQSTM1/p62 protein in Nrf2/Keap1 signalling and autophagy^(^ ^36^ ^)^. Moreover, modulation of expression of visfatin, *sirtuin-1* and GLUT (2 and 4) may improve glucose metabolism by suppressing oxidative stress and increasing potential to internalise glucose by extrahepatic tissues^(^ ^37^ ^)^. Besides the antioxidant effects, stilbenes have also been suggested to regulate circulating leptin levels and improve insulin signalling, both implicated in type 2 diabetes risk^(^ ^38^ ^)^. However, the mechanisms of action for the hypothesised effects are by far complex and the knowledge on them is still evolving.

In the present study, no significant results were found for phyto-oestrogens, such as isoflavones and lignans. In our previous cross-sectional analysis, we found some positive association between impaired glucose metabolism and intake of isoflavones, which have a weak oestrogenic action. These findings are substantially in contrast with other recent studies showing that urinary excretion of isoflavones and lignans or markers of consumption were associated with lower risk of type 2 diabetes^(^ ^39^ ^,^ ^40^ ^)^. However, other epidemiological studies reported contrasting results^(^ ^41^ ^)^ and analyses of large European and US cohorts showed no association of soya food intake, isoflavones and lignans and risk of type 2 diabetes^(^ ^42^ ^,^ ^43^ ^)^. Consumption of soya foods in our cohort was very low and main food sources of phyto-oestrogens in non-Asian population are generally legumes, whole wheat products and seeds^(^ ^44^ ^)^. Clinical trials on flaxseeds and lignans and glycaemic control showed similar contrasting results^(^ ^45^ ^,^ ^46^ ^)^. A meta-analysis of twenty-four intervention studies on soy and isoflavone intake and glycaemic control showed no significant effect on fasting glucose and insulin of eight trials with isoflavone extract and six studies with isolated soya protein, but reduction in fasting glucose concentrations in nine studies that used whole soya foods or soya diets (suggesting that components other than phyto-oestrogens may be responsible for the potential benefits)^(^ ^47^ ^)^. Among other issues related to the contrasting results on phytoestrogens has been pointed out the large inter-individual variation in plasma concentrations of isoflavones, which may limit the use of intake data for risk assessment^(^ ^48^ ^)^. Phyto-oestrogens have been hypothesised to ameliorate glucose metabolism and prevent type 2 diabetes by decreasing the activity of key enzymes related to blood glucose and HbA1c levels (including hepatic glucose-6-phosphatase and phosphoenolpyruvate carboxykinase, fatty acid synthase, *β*-oxidation and carnitine palmitoyltransferase) and activating the PPAR gene expression pathway^(^ ^49^ ^)^. However, given the aforementioned issues, further studies are needed to provide stronger evidence of the biological rationale.

Other limitations should be taken into account when considering results from this study. First, the observational design of the study can only partially provide evidence of causation. Second, due to relatively low response rate in the follow-up examination, number of participants included to the analysis was substantially lower than screened at baseline. This influenced the representativeness of the sample negatively, but there is evidence that low participation rate affect less the relations studied^(^ ^50^ ^,^ ^51^ ^)^. Nevertheless, it could contribute to an underestimation of the associations found because of the higher no-participation in diabetics. Third, as information on diet was collected at baseline only, we were unable to test whether dietary habits had changed during follow-up. However, during 4 years it is unlikely that there was a major change in dietary habits of the population. We also acknowledge that our assumption on the induction period between nutritional exposure to polyphenols and T2D might have been too short. In any case, the FFQ usually captures the habitual diet and may represent a proxy for long-term dietary exposures. The potentially unrealistic assumed induction period remains a limitation of our study, and it more probably would have produced and underestimation of the association. Fourth, although sensitivity of diabetes self-reports is high (about 70 %)^(^ ^52^ ^)^, there is a small proportion of individuals that are unaware of their disease. If we consider that diabetics could have dropped the study to a larger extent than non-diabetics, this causes that the ascertained number of cases would be under-estimated, thus reducing the statistical power of the analyses. However, given the prospective design of the study, it is unlikely that this under-recording of cases would be differential according to the exposure, thus affecting the precision, but not the validity of the results. However, such issues are common to all previous studies using the same methodology and as polyphenol exposure was ascertained before diagnosis of disease, misclassification would tend to bias estimates toward the null and underestimate true associations. Fifth, some polyphenol-rich foods, such as herbs and spices, were not included in FFQ, which might lead to underestimation of the exposure. Nevertheless we don’t expect that contribution of polyphenols from spices and herbs in the total polyphenols intake is high enough to materially affect the results. Fifth, use of table content databases would have inevitably led to some misclassification of polyphenol intake. Sixth, consumption of some polyphenol classes (i.e. stilbenes, lignans and isoflavones) in ‘normal’ diets can be negligible, and consequently difficult to be accurately estimated. Despite we found significant results for such polyphenol groups, it should be considered that amount consumed was very low. Seventh, despite we adjusted the analyses for a number of potential confounders, other untested variables (i.e. family history of diabetes) may contribute to residual confounding.

In conclusion, higher intake of a variety of polyphenols may decrease the risk of type 2 diabetes. Besides the fact that results from this study may not be generalisable to other populations, they provide further evidence that potential association between polyphenols intake and cardiovascular risk factors may exist. Our study added to the current knowledge further information regarding other polyphenol classes potentially explaining the aforementioned contrasting results. Limitation of previous studies investigating individual classes of polyphenols may have led to underestimation of their effects by not taking into account possible interactions. Further studies with experimental design are needed to establish the effects that can specifically attributed to each polyphenol class in order to identify specific polyphenol-rich foods that may contribute to the prevention of cardiovascular risk factors.

## Supplementary material

For supplementary material accompanying this paper visit http://dx.doi.org/10.1017//S0007114517001805. click here to view supplementary material
