# Supplementary material for: Dietary polyphenol intake and risk of type 2 diabetes in the Polish arm of the Health, Alcohol and Psychosocial factors in Eastern Europe (HAPIEE) study
Source: Br J Nutr. 2017 Jul 14;118(1):60–8. doi: 10.1017/S0007114517001805 (PMC5565930; doi:10.1017/S0007114517001805)
Supplement: Supplementary file 1 [file S0007114517001805sup001.docx]

Supplementary Table 1. Major food sources of total and individual classes and subclasses of polyphenols.

| Polyphenol class | Main food contributors (% contribution to polyphenol class) | | |
| --- | --- | --- | --- |
| Total polyphenols | Coffee (40) | Tea (27) | Chocolate (8) |
| Flavonoids | Tea (48) | Chocolate (18) | Apples (8) |
| Anthocyanins | Black currant (21) | Beans (19) | Strawberries (16) |
| Dihydrochalcones | Apple (93) | Apple juice (7) |  |
| Flavanols | Tea (60) | Chocolate (25) | Apples (7) |
| Flavanones | Orange juice (29) | Squash (24) | Oranges (23) |
| Flavones | Flour (51) | Orange juice (23) | Squash (10) |
| Flavonols | Tea (47) | Onion (13) | Spinach (13) |
| Isoflavonoids | Soy meat (85) | Beans (12) | Soy milk (3) |
| Phenolic acids | Coffee (66) | Tea (12) | Vegetable oils (7) |
| Hydroxybenzoic acids | Tea (89) | Apples (3) | Raspberries (2) |
| Hydroxycinnamic acids | Coffee (75) | Vegetable oil (8) | Apples (5) |
| Lignans | Seeds (51) | Tea (27) | Dark bread (8) |
| Stilbenes | Red wine (56) | Strawberries (14) | White wine (12) |
| Others | Beer (33) | Cereals (7) | Coffee (3) |

Supplementary Table 2. Odds ratios (ORs) and 95% confidence intervals (CIs) for the association between cumulative polyphenol intake (total and main groups) and type 2 diabetes.

|  | Polyphenol quartiles, Men | | | | | | | |  | Polyphenol quartiles, women | | | | | | | |
| --- | --- | --- | --- | --- | --- | --- | --- | --- | --- | --- | --- | --- | --- | --- | --- | --- | --- |
|  | Q1 | | Q2 | | Q3 | | Q4 | |  | Q1 | | Q2 | | Q3 | | Q4 | |
| Total polyphenols, mean (SD), mg/d | 1029.4 | 204.4 | 1469.4 | 100.5 | 1872.3 | 134.9 | 2661.0 | 666.0 |  | 1024.3 | 219.0 | 1469.8 | 103.7 | 1872.9 | 138.5 | 2605.4 | 548.1 |
| No. of cases | 89 | 13.8 | 55 | 8.4 | 44 | 6.3 | 31 | 4.2 |  | 78 | 11.1 | 66 | 8.3 | 47 | 6.0 | 46 | 5.8 |
| Model 1^a^ | 1 |  | 0.62 | 0.43, 0.89 | 0.49 | 0.33, 0.73 | 0.35 | 0.22, 0.55 |  | 1 |  | 0.79 | 0.56, 1.13 | 0.60 | 0.41, 0.89 | 0.64 | 0.42, 0.96 |
| Model 2 ^b^ | 1 |  | 0.72 | 0.49, 1.08 | 0.38 | 0.23, 0.61 | 0.31 | 0.18, 0.52 |  | 1 |  | 0.78 | 0.53, 1.15 | 0.55 | 0.36, 0.86 | 0.53 | 0.33, 0.85 |
| Total flavonoids, mean (SD), mg/d | 505.3 | 121.8 | 754.9 | 56.4 | 962.7 | 69.8 | 1462.8 | 570.1 |  | 498.9 | 127.8 | 757.7 | 54.8 | 954.5 | 69.6 | 1441.9 | 441.4 |
| No. of cases | 87 | 13.7 | 58 | 8.6 | 41 | 6.1 | 33 | 4.4 |  | 78 | 10.2 | 64 | 8.5 | 51 | 6.3 | 44 | 5.8 |
| Model 1^a^ | 1 |  | 0.63 | 0.44, 0.91 | 0.47 | 0.31, 0.70 | 0.38 | 0.24, 0.60 |  | 1 |  | 0.91 | 0.64, 1.30 | 0.71 | 0.48, 1.04 | 0.73 | 0.48, 1.11 |
| Model 2 ^b^ | 1 |  | 0.61 | 0.40, 0.91 | 0.38 | 0.23, 0.61 | 0.30 | 0.17, 0.52 |  | 1 |  | 0.90 | 0.61, 1.33 | 0.61 | 0.40, 0.94 | 0.59 | 0.36, 0.96 |
| Phenolic acids, mean (SD), mg/d | 286.6 | 85.1 | 613.0 | 63.1 | 835.0 | 165.6 | 1504.5 | 330.0 |  | 290.4 | 86.0 | 618.1 | 58.5 | 824.3 | 160.6 | 1462.2 | 266.6 |
| No. of cases | 61 | 8.7 | 74 | 11.5 | 47 | 6.8 | 37 | 5.3 |  | 65 | 9.4 | 63 | 8.0 | 67 | 8.4 | 42 | 5.3 |
| Model 1^a^ | 1 |  | 1.31 | 0.92, 1.88 | 0.89 | 0.59, 1.33 | 0.68 | 0.44, 1.04 |  | 1 |  | 0.83 | 0.57, 1.19 | 1.01 | 0.70, 1.46 | 0.61 | 0.41, 0.92 |
| Model 2 ^b^ | 1 |  | 1.41 | 0.94, 2.11 | 0.86 | 0.54, 1.35 | 0.56 | 0.33, 0.94 |  | 1 |  | 0.94 | 0.63, 1.40 | 0.98 | 0.65, 1.48 | 0.63 | 0.40, 1.00 |
| Stilbenes, mean (SD), mg/d | 0.005 | 0.003 | 0.017 | 0.005 | 0.046 | 0.013 | 0.590 | 1.281 |  | 0.004 | 0.003 | 0.016 | 0.005 | 0.046 | 0.013 | 0.606 | 1.384 |
| No. of cases | 84 | 14.0 | 43 | 6.4 | 57 | 8.2 | 35 | 4.6 |  | 85 | 11.8 | 59 | 8.0 | 50 | 6.3 | 43 | 5.2 |
| Model 1^a^ | 1 |  | 0.48 | 0.32, 0.71 | 0.66 | 0.46, 0.95 | 0.35 | 0.23, 0.53 |  | 1 |  | 0.73 | 0.51, 1.04 | 0.59 | 0.40, 0.86 | 0.46 | 0.32, 0.68 |
| Model 2 ^b^ | 1 |  | 0.48 | 0.30, 0.74 | 0.64 | 0.42, 0.97 | 0.27 | 0.16, 0.47 |  | 1 |  | 0.70 | 0.47, 1.02 | 0.46 | 0.30, 0.71 | 0.41 | 0.25, 0.67 |
| Lignans, mean (SD), mg/d | 0.1 | 0.0 | 0.2 | 0.0 | 0.3 | 0.0 | 1.6 | 22.4 |  | 0.1 | 0.0 | 0.2 | 0.0 | 0.3 | 0.0 | 1.1 | 16.1 |
| No. of cases | 63 | 9.3 | 41 | 6.3 | 55 | 8.4 | 60 | 8.0 |  | 58 | 7.4 | 62 | 8.2 | 59 | 7.5 | 58 | 7.8 |
| Model 1^a^ | 1 |  | 0.74 | 0.49, 1.12 | 1.05 | 0.71, 1.54 | 1.15 | 0.78, 1.69 |  | 1 |  | 1.25 | 0.86, 1.83 | 1.28 | 0.81, 1.74 | 1.37 | 0.92, 2.03 |
| Model 2 ^b^ | 1 |  | 0.68 | 0.41, 1.10 | 1.04 | 0.67, 1.61 | 1.02 | 0.66, 1.60 |  | 1 |  | 1.32 | 0.86, 2.01 | 1.26 | 0.88, 1.94 | 1.32 | 0.85, 2.05 |
| ^a^ Adjusted for age (continuous) and total energy intake (continuous).  ^b^ Model 1 + adjusted for body mass index (continuous), physical activity (low/medium/high), educational status (low/medium/high), smoking status (yes/no), alcohol consumption (yes/no), alcohol intake (continuous), menopausal status (women only, yes/no), and dietary fiber (continuous). | | | | | | | | | | | | | | | | | |

Supplementary Table 3. Odds ratios (ORs) and 95% confidence intervals (CIs) for the association between phenolic acid subclasses and type 2 diabetes.

|  | Men  Polyphenol quartiles | | | | | | | |  | Women  Polyphenol quartiles | | | | | | | |
| --- | --- | --- | --- | --- | --- | --- | --- | --- | --- | --- | --- | --- | --- | --- | --- | --- | --- |
|  | Q1 | | Q2 | | Q3 | | Q4 | |  | Q1 | | Q2 | | Q3 | | Q4 | |
| Hydroxybenzoic acids, mean (SD), mg/d | 43.2 | 23.9 | 85.7 | 0.9 | 91.7 | 3.7 | 156.7 | 30.4 |  | 192.9 | 74.0 | 532.6 | 64.1 | 722 .1 | 167.5 | 1367.3 | 258.5 |
| No. of cases | 65 | 9.9 | 56 | 8.6 | 57 | 8.3 | 41 | 5.6 |  | 59 | 7.8 | 62 | 7.8 | 71 | 9.2 | 45 | 6.0 |
| Model 1^a^ | 1 |  | 0.85 | 0.58, 1.23 | 0.97 | 0.66, 1.42 | 0.63 | 0.42, 0.96 |  | 1 |  | 1.03 | 0.71, 1.50 | 1.46 | 1.01, 2.13 | 0.88 | 0.59, 1.33 |
| Model 2 ^b^ | 1 |  | 0.98 | 0.63, 1.52 | 0.99 | 0.63, 1.55 | 0.58 | 0.35, 0.94 |  | 1 |  | 1.11 | 0.73, 1.67 | 1.35 | 0.89, 2.06 | 0.83 | 0.53, 1.31 |
| Hydroxycinnamic acids, mean (SD), mg/d | 183.4 | 73.0 | 528.2 | 69.2 | 727.3 | 170.5 | 1408.3 | 324.8 |  | 43.7 | 23.1 | 85.7 | 0.9 | 91.7 | 3.8 | 157.6 | 32.5 |
| No. of cases | 61 | 8.7 | 65 | 10.1 | 55 | 8.1 | 38 | 5.4 |  | 66 | 9.5 | 63 | 8.2 | 66 | 8.1 | 42 | 5.3 |
| Model 1^a^ | 1 |  | 1.11 | 0.77, 1.61 | 1.08 | 0.73, 1.59 | 0.69 | 0.45, 1.06 |  | 1 |  | 0.82 | 0.57, 1.18 | 0.97 | 0.67, 1.39 | 0.60 | 0.40, 0.90 |
| Model 2 ^b^ | 1 |  | 1.28 | 0.84, 1.94 | 1.04 | 0.66, 1.62 | 0.59 | 0.35, 0.99 |  | 1 |  | 0.95 | 0.64, 1.43 | 0.97 | 0.64, 1.46 | 0.63 | 0.40, 0.99 |
| ^a^ Adjusted for age (continuous) and total energy intake (continuous).  ^b^ Model 1 + adjusted for body mass index (continuous), physical activity (low/medium/high), educational status (low/medium/high), smoking status (yes/no), alcohol consumption (yes/no), alcohol intake (continuous), menopausal status (women only, yes/no), and dietary fiber (continuous). | | | | | | | | | | | | | | | | | |

Supplementary Table 3. Odds ratios (ORs) and 95% confidence intervals (CIs) for the association between flavonoid subclasses and type 2 diabetes.

|  | Men  Polyphenol quartiles | | | | | | | |  | Women  Polyphenol quartiles | | | | | | | |
| --- | --- | --- | --- | --- | --- | --- | --- | --- | --- | --- | --- | --- | --- | --- | --- | --- | --- |
|  | Q1 | | Q2 | | Q3 |  | Q4 | |  | Q1 | | Q2 | | Q3 | | Q4 | |
| Flavanols, mean (SD), mg/d | 327.7 | 108.7 | 516.9 | 56.5 | 683.9 | 44.0 | 1122.6 | 523.0 |  | 319.8 | 111.0 | 521.9 | 57.3 | 679.8 | 43.6 | 1077.6 | 324.6 |
| No. of cases | 86 | 12.8 | 61 | 9.5 | 35 | 5.2 | 37 | 4.9 |  | 65 | 9.0 | 78 | 9.7 | 51 | 6.5 | 43 | 5.6 |
| Model 1^a^ | 1 |  | 0.77 | 0.54, 1.09 | 0.43 | 0.29, 0.66 | 0.45 | 0.30, 0.69 |  | 1 |  | 1.24 | 0.87, 1.76 | 0.85 | 0.57, 1.26 | 0.79 | 0.52, 1.21 |
| Model 2 ^b^ | 1 |  | 0.76 | 0.51, 1.15 | 0.42 | 0.26, 0.68 | 0.40 | 0.24, 0.66 |  | 1 |  | 1.04 | 0.70, 1.54 | 0.78 | 0.50, 1.20 | 0.70 | 0.44, 1.12 |
| Flavonols, mean (SD), mg/d | 60.5 | 14.5 | 89.7 | 6.4 | 114.0 | 7.7 | 161.6 | 33.4 |  | 59.8 | 14.9 | 89.5 | 6.3 | 113.2 | 7.6 | 164.9 | 55.7 |
| No. of cases | 62 | 9.7 | 54 | 8.1 | 51 | 7.3 | 52 | 7.2 |  | 62 | 8.0 | 60 | 7.9 | 59 | 7.7 | 56 | 7.3 |
| Model 1^a^ | 1 |  | 0.89 | 0.61, 1.31 | 0.86 | 0.58, 1.27 | 1.01 | 0.67, 1.52 |  | 1 |  | 1.12 | 0.77, 1.64 | 1.19 | 0.81, 1.74 | 1.23 | 0.83, 1.84 |
| Model 2 ^b^ | 1 |  | 0.85 | 0.55, 1.34 | 0.77 | 0.49, 1.23 | 0.72 | 0.43, 1.21 |  | 1 |  | 1.09 | 0,71, 1.67 | 1.16 | 0.75, 1.78 | 1.01 | 0.63, 1.62 |
| Flavanones, mean (SD), mg/d | 24.5 | 11.4 | 59.4 | 10.9 | 105.3 | 16.0 | 219.8 | 88.4 |  | 24.7 | 11.4 | 59.8 | 11.2 | 105.8 | 15.6 | 217.9 | 90.8 |
| No. of cases | 57 | 9.1 | 65 | 9.5 | 58 | 8.2 | 39 | 5.4 |  | 80 | 10.9 | 51 | 6.3 | 55 | 7.3 | 51 | 6.6 |
| Model 1^a^ | 1 |  | 0.83 | 0.57, 1.21 | 0.81 | 0.55, 1.20 | 0.75 | 0.50, 1.13 |  | 1 |  | 1.01 | 0.71, 1.43 | 0.76 | 0.51, 1.12 | 0.95 | 0.65, 1.40 |
| Model 2 ^b^ | 1 |  | 0.70 | 0.45, 1.04 | 0.71 | 0.46, 1.11 | 0.65 | 0.40, 1.03 |  | 1 |  | 1.07 | 0.73, 1.58 | 0.73 | 0.47, 1.13 | 0.84 | 0.54, 1.30 |
| Flavones, mean (SD), mg/d | 1.9 | 0.7 | 4.2 | 0.7 | 7.9 | 1.5 | 16.6 | 7.5 |  | 1.9 | 0.7 | 4.3 | 0.7 | 7.9 | 1.5 | 16.4 | 7.8 |
| No. of cases | 64 | 10.6 | 72 | 10.2 | 49 | 6.7 | 34 | 4.9 |  | 80 | 10.9 | 51 | 6.3 | 55 | 7.3 | 51 | 6.6 |
| Model 1^a^ | 1 |  | 1.05 | 0.73, 1.50 | 0.72 | 0.48, 1.08 | 0.60 | 0.38, 0.94 |  | 1 |  | 0.61 (0.42, 0.88) | 0.42, 0.88 | 0.77 | 0.53, 1.11 | 0.77 | 0.52, 1.14 |
| Model 2 ^b^ | 1 |  | 0.90 | 0.59, 1.37 | 0.61 | 0.38, 0.97 | 0.41 | 0.23, 0.72 |  | 1 |  | 0.57 | 0.37, 0.85 | 0.69 | 0.45, 1.04 | 0.52 | 0.32, 0.85 |
| Anthocyanins, mean (SD), mg/d | 4.3 | 2.0 | 8.7 | 1.2 | 14.4 | 2.4 | 81.9 | 161.7 |  | 4.4 | 1.9 | 8.7 | 1.2 | 14.5 | 2.4 | 100.9 | 204.5 |
| No. of cases | 57 | 9.1 | 65 | 9.5 | 58 | 8.2 | 39 | 5.4 |  | 57 | 7.6 | 76 | 10.1 | 55 | 7.0 | 49 | 6.2 |
| Model 1^a^ | 1 |  | 1.16 | 0.80, 1.69 | 1.06 | 0.72, 1.57 | 0.76 | 0.49, 1.17 |  | 1 |  | 1.48 | 1.03, 2.13 | 1.09 | 0.74, 1.62 | 1.00 | 0.67, 1.51 |
| Model 2 ^b^ | 1 |  | 0.99 | 0.64, 1.53 | 0.78 | 0.49, 1.24 | 0.59 | 0.35, 1.00 |  | 1 |  | 1.37 | 0.92, 20.5 | 0.94 | 0.60, 1.46 | 0.77 | 0.47, 1.26 |
| Isoflavones, mean (SD), mg/d | 0.0005 | 0.0018 | 0.1406 | 0.0017 | 0.1961 | 0.0001 | 4.5715 | 16.7192 |  | 0.0003 | 0.0011 | 0.1403 | 0.010 | 0.1960 | 0.0001 | 5.2629 | 9.532 |
| No. of cases | 41 | 10.3 | 62 (7.6) | 7.6 | 64 | 9.0 | 52 | 6.5 |  | 33 | 7.3 | 83 | 8.4 | 67 | 7.9 | 54 | 6.9 |
| Model 1^a^ | 1 |  | 0.71 | 0.47, 1.08 | 0.93 | 0.61, 1.41 | 0.72 | 0.47, 1.12 |  | 1 |  | 1.15 | 0.76, 1.76 | 1.19 | 0.77, 1.85 | 1.11 | 0.71, 1.76 |
| Model 2 ^b^ | 1 |  | 0.66 | 0.40, 1.08 | 0.87 | 0.53, 1.41 | 0.74 | 0.44, 1.23 |  | 1 |  | 1.19 | 0.75, 1.89 | 1.15 | 0.70, 1.86 | 1.14 | 0.69, 1.90 |
| Dihydrochalcones, mean (SD), mg/d | 1.9 | 1.2 | 6.8 | 1.7 | 9.7 | 1.1 | 25.1 | 7.8 |  | 1.9 | 1.2 | 6.9 | 1.6 | 9.7 | 1.0 | 24.9 | 7.6 |
| No. of cases | 47 | 7.0 | 71 | 10.1 | 29 | 4.9 | 72 | 9.5 |  | 47 | 7.2 | 59 | 7.6 | 59 | 7.4 | 72 | 8.5 |
| Model 1^a^ | 1 |  | 1.55 | 1.05, 2.28 | 0.81 | 0.50, 1.31 | 1.72 | 1.16, 2.55 |  | 1 |  | 1.14 | 0.77, 1.71 | 1.26 | 0.84, 1.89 | 1.53 | 1.03, 2.27 |
| Model 2 ^b^ | 1 |  | 1.22 | 0.79, 1.88 | 0.58 | 0.33, 1.03 | 1.10 | 0.67, 1.78 |  | 1 |  | 0.92 | 0.59, 1.44 | 1.03 | 0.65, 1.62 | 1.16 | 0.73, 1.84 |
| ^a^ Adjusted for age (continuous) and total energy intake (continuous).  ^b^ Model 1 + adjusted for body mass index (continuous), physical activity (low/medium/high), educational status (low/medium/high), smoking status (yes/no), alcohol consumption (yes/no), alcohol intake (continuous), menopausal status (women only, yes/no), and dietary fiber (continuous). | | | | | | | | | | | | | | | | | |

Supplementary Table 4. Odds ratios (ORs) and 95% confidence intervals (CIs) for the association between other polyphenols and type 2 diabetes.

|  | Polyphenol quartiles, Men | | | | | | | |  | Polyphenol quartiles, women | | | | | | | |
| --- | --- | --- | --- | --- | --- | --- | --- | --- | --- | --- | --- | --- | --- | --- | --- | --- | --- |
|  | Q1 | | Q2 | | Q3 | | Q4 | |  | Q1 | | Q2 | | Q3 | | Q4 | |
| Others, mean (SD), mg/d | 6.1 | 3.1 | 16.7 | 3.0 | 33.2 | 5.6 | 76.4 | 38.9 |  | 6.2 | 2.9 | 16.8 | 3.1 | 32.7 | 5.6 | 76.9 | 36.7 |
| No. of cases | 47 | 6.9 | 46 | 6.8 | 65 | 9.7 | 61 | 8.7 |  | 85 | 11.8 | 59 | 8.0 | 50 | 6.3 | 43 | 5.2 |
| Model 1 ^a^ | 1 |  | 1.02 | 0.67, 1.56 | 1.55 | 1.04, 2.29 | 1.60 | 1.07, 2.40 |  | 1 |  | 0.85 | 0.58, 1.24 | 0.92 | 0.63, 1.33 | 0.97 | 0.66, 1.43 |
| Model 2 ^b^ | 1 |  | 1.19 | 0.74, 1.89 | 1.39 | 0.88, 2.18 | 1.21 | 0.76, 1.92 |  | 1 |  | 0.97 | 0.64, 1.47 | 0.85 | 0.55, 1.30 | 0.91 | 0.58, 1.41 |
| ^a^ Adjusted for age (continuous) and total energy intake (continuous).  ^b^ Model 1 + adjusted for body mass index (continuous), physical activity (low/medium/high), educational status (low/medium/high), smoking status (yes/no), alcohol consumption (yes/no), alcohol intake (continuous), menopausal status (women only, yes/no), and dietary fiber (continuous). | | | | | | | | | | | | | | | | | |
